# Media*Dive*: the expert-curated cultivation media database

**DOI:** 10.1093/nar/gkac803

**Published:** 2022-09-22

**Authors:** Julia Koblitz, Philipp Halama, Stefan Spring, Vera Thiel, Christiane Baschien, Richard L Hahnke, Michael Pester, Jörg Overmann, Lorenz Christian Reimer

**Affiliations:** Leibniz Institute DSMZ-German Collection of Microorganisms and Cell Cultures, Braunschweig, Germany; Leibniz Institute DSMZ-German Collection of Microorganisms and Cell Cultures, Braunschweig, Germany; Leibniz Institute DSMZ-German Collection of Microorganisms and Cell Cultures, Braunschweig, Germany; Leibniz Institute DSMZ-German Collection of Microorganisms and Cell Cultures, Braunschweig, Germany; Leibniz Institute DSMZ-German Collection of Microorganisms and Cell Cultures, Braunschweig, Germany; Leibniz Institute DSMZ-German Collection of Microorganisms and Cell Cultures, Braunschweig, Germany; Leibniz Institute DSMZ-German Collection of Microorganisms and Cell Cultures, Braunschweig, Germany; Technical University of Braunschweig, Institute for Microbiology, Braunschweig, Germany; Leibniz Institute DSMZ-German Collection of Microorganisms and Cell Cultures, Braunschweig, Germany; Technical University of Braunschweig, Institute for Microbiology, Braunschweig, Germany; Leibniz Institute DSMZ-German Collection of Microorganisms and Cell Cultures, Braunschweig, Germany

## Abstract

We present Media*Dive* (https://mediadive.dsmz.de), a comprehensive and expert-curated cultivation media database, which comprises recipes, instructions and molecular compositions of >3200 standardized cultivation media for >40 000 microbial strains from all domains of life. Media*Dive* is designed to enable broad range applications from every-day-use in research and diagnostic laboratories to knowledge-driven support of new media design and artificial intelligence-driven data mining. It offers a number of intuitive search functions and comparison tools, for example to identify media for related taxonomic groups and to integrate strain-specific modifications. Besides classical PDF archiving and printing, the state-of-the-art website allows paperless use of media recipes on mobile devices for convenient wet-lab use. In addition, data can be retrieved using a RESTful web service for large-scale data analyses. An internal editor interface ensures continuous extension and curation of media by cultivation experts from the Leibniz Institute DSMZ, which is interlinked with the growing microbial collections at DSMZ. External user engagement is covered by a dedicated media builder tool. The standardized and programmatically accessible data will foster new approaches for the design of cultivation media to target the vast majority of uncultured microorganisms.

## INTRODUCTION

Our views on microbial biodiversity have evolved dramatically in recent decades, mainly due to rapid advances in sequencing technologies applied in 16S/ITS rRNA gene amplicon sequencing and metagenomics. However, we are still unable to cultivate representative species of the majority of phylogenetic lineages (1). Accordingly, most of what we know about microorganisms either derives from the minority of well-studied model organisms, or has been inferred from sequenced genomes. Although we can generate a wealth of information through the latter (2–4), it is essential to culture microorganisms in the laboratory, to prove or falsify predictions from genome analyses, to discover and elucidate new metabolic pathways and to better understand their ecological niches (5).

In this regard, the development of novel types of cultivation media and the subsequent establishment of pure cultures represent a key challenge (6,7). Despite their fundamental importance, cultivation media as data resource are mostly neglected, especially in terms of standardization and availability. Recipes themselves are mostly found as unstandardized documents that are difficult to screen, cannot be linked to other data, and from which quantitative analyses can only be conducted with great effort.

There have been previous efforts to standardize cultivation media and make them globally available. For instance, MediaDB (8) has compiled the molecular composition of 471 defined media (https://mediadb.systemsbiology.net/). Primarily made for systems biology, MediaDB focused on model organisms and therefore is limited to a list of 210 microbial strains representing only 59 species. The KOMODO database (9) builds upon 1300 media recipes from the DSMZ digitized in 2015. The authors of this study mapped media ingredients to the SEED database, and presented them on their website (https://komodo.modelseed.org). In addition, they provided data for about 18,000 microbial species that grow on the media. Both approaches specifically target systems biology analyses by focusing on the final media composition and not the full and detailed description how to prepare a respective medium including standardized base constituents (e.g. trace element and vitamin solutions) and special knowledge-requiring intermediate steps. Moreover, both databases are not maintained anymore with the latest update being 7–8 years old.

Meanwhile, the number of cultivation media listed with the DSMZ has increased to nearly 1850 and the cultivation experts of the DSMZ have updated many existing recipes. Building on this new but unstructured knowledge, we digitized the complete DSMZ media list by developing a highly standardized data structure not only for the media constituents but also for their preparation. This has resulted in the currently most extensive and comprehensive database for cultivation media, which we present in this contribution.

## CONTENT

Media*Dive* is the world's first cultivation media database that provides molecular compositions and growth data as well as recipes and cultivation instructions. All information is based on the long-term curated knowledge base of DSMZ cultivation experts. In addition, the database extends from DSMZ media to media collections from other resources, e.g. the Japanese collection of microorganisms JCM. Media*Dive* targets a broad range of user groups: from scientists developing a new medium over technical assistants aiming to cultivate a particular microorganism in research, development, or diagnostic laboratories, to bioinformaticians conducting large-scale data mining. All cultivation media are presented together with a set of intuitive search functions on a responsive state-of-the-art website. Most importantly and in contrast to previous digitization projects for cultivation media, we provide a continuous support, development and curation of the Media*Dive* database. This long-term engagement is interlinked with the permanent microbial collections of DSMZ and based on an internal curation interface that was developed to allow live editing of media by DSMZ cultivation experts. To expand this engagement to the whole scientific community, any user can design an own medium using our medium builder tool and make it available to the public.

The core of Media*Dive* are 3158 cultivation media recipes that contain 5222 distinct solutions and 1193 different ingredients. In addition to the recipes, the database provides important metadata on the media, including, but not limited to final pH, gas phases, equipment needed, and molecular compositions of the final medium. The media data are complemented with growth data for 41,626 microbial strains. This includes >32 000 bacteria and archaea, 10 000 fungi including yeasts, and hosts of 500 phages. Since some of these strains require adjustments to the medium for proper growth, there is a total of 4073 different medium compositions in the database. The media recipes originated from two of the largest culture collections worldwide, 1845 media from the DSMZ and 1313 media from the JCM. They have been extracted from non-standardized formats (PDF, Word, HTML) in a semi-automatic approach, followed by extensive manual curation.

The DSMZ cultivation media data in Media*Dive* are live-curated, i.e. they are continuously maintained by cultivation experts of the DSMZ. For this purpose, we developed a complete web-based curation interface that is used to add new cultivation media, edit existing recipes and add also growth data sets that describe whether growth occurs on a given medium and at which growth rates. The live-curated data are complemented with integrated external data sets, link-outs and identifiers from other databases. A large data set from the Media*Dive* sibling database Bac*Dive* (10) was integrated to give an overview on phenotypic traits, e.g. temperature optimum and oxygen tolerance, of all strains that are known to grow on a medium. These phenotype data are shown on the medium overview page and include data on pH, nutrition, oxygen, salt, and temperature requirements. Data on nomenclature provided by Media*Dive* is updated on a regular basis using correct names from List of Prokaryotic names with Standing in Nomenclature (LPSN) (11) to support a taxonomy-based media search that we discuss further below. Ingredients were standardized and extensively complemented with external data, such as identifiers from other databases, chemical information (sum formula, molecular mass, density), and compound synonyms from eight different online resources (Table 1).

**Table 1.** Resources that have been mapped to ingredients of Media*Dive*

Resource	# of ingredients	Web address	Ref.
PubChem	616	pubchem.ncbi.nlm.nih.gov	(12)
ChEBI	687	ebi.ac.uk/chebi	(13)
KEGG	315	genome.jp/dbget-bin	(14)
BRENDA	226	brenda-enzymes.org	(15)
MetaCyc	237	metacyc.org	(16)
SABIO-RK	169	sabio.h-its.org	(17)
CAS Common Chemistry®	867	commonchemistry.cas.org	(18)
GESTIS ZVG	418	gestis.dguv.de	(19)

## IMPLEMENTATION

Media*Dive* is accessible from every modern browser and is free of charge. To enhance the accessibility by search engines, we added *schema.org* and *bioschemas.org* profiles. We included the MolecularEntity profile for ingredients, the LabProtocol profile for solution recipes, the DataSet profile for culture media entries, and the DataCatalog profile for Media*Dive* in general.

The backend is completely written in PHP (v7.4.19). Since Media*Dive* is on one hand a comprehensive external resource but serves on the other hand as an internal curation interface, we developed our own router class. The frontend relies on JavaScript and jQuery. The relational database engine is MariaDB (v10.5.13). The database scheme consists of 19 tables, whereof five tables (media, solutions, ingredients, strains and modifications) build the core of the database with all other tables being connected to them.

## USER INTERFACE

Media*Dive* features a user-friendly, clearly structured and evident interface. Content pages and tools can be reached from a side navigation that can be hidden to make more space for displaying data on the screen. The header navigation has a breadcrumb menu that gives an overview on the current location, as well as a search bar that allows fast media look-up and features auto-suggestions.

Media*Dive* is completely mobile-friendly and parts of the website are exclusively designed for mobile devices, e.g. the *medium cooking guide* that allows medium preparation in the wet-lab without printing the recipe.

There are many ways to receive help directly on the page. Media*Dive* has a comprehensive documentation, including detailed explanations of important web pages, alongside with cultivation hints and other information. Many pages also feature an interactive tour (by clicking on the *Guided tour* button in the top-right corner), that explains important parts of the page. Finally, we have recorded video tutorials that give an overview on the page or guide the user through more complex topics, e.g. strain modifications.

### Content pages

Six content pages allow targeted browsing of the data and provide a number of search and filter functions. The media page supports the user in finding its cultivation medium of interest, either by name (including synonyms) or medium number. Cultivation media can be filtered by organism group, complexity, source, or final pH. It is also possible to define favorite cultivation media that are saved in the browser (cookie) and can thus be used for filtering. To search for media based on their components, there is an extra tool called medium finder, which is discussed below. Solutions can also be filtered by name or internal database ID. Ingredients can be searched by name, internal database ID and CAS registry number. Additionally, ingredients were aggregated into a total of 775 ingredient groups. These groups were manually assigned to take synonyms and rather similar ingredients into account when using various functions, e.g. glucose, (+)-glucose and beta-glucose are treated the same when using the medium finder. Strains can be filtered by media they grow on and searched by species names, as far as they have been classified yet, as well as culture collection numbers. Species nomenclature depends on the providers of the data and is updated regularly. Steps provide a full text search and filters for standardized steps. The gas phase page supports a search function to define minimal and maximal values for each gas constituent.

### The medium viewer

**Figure 1.**
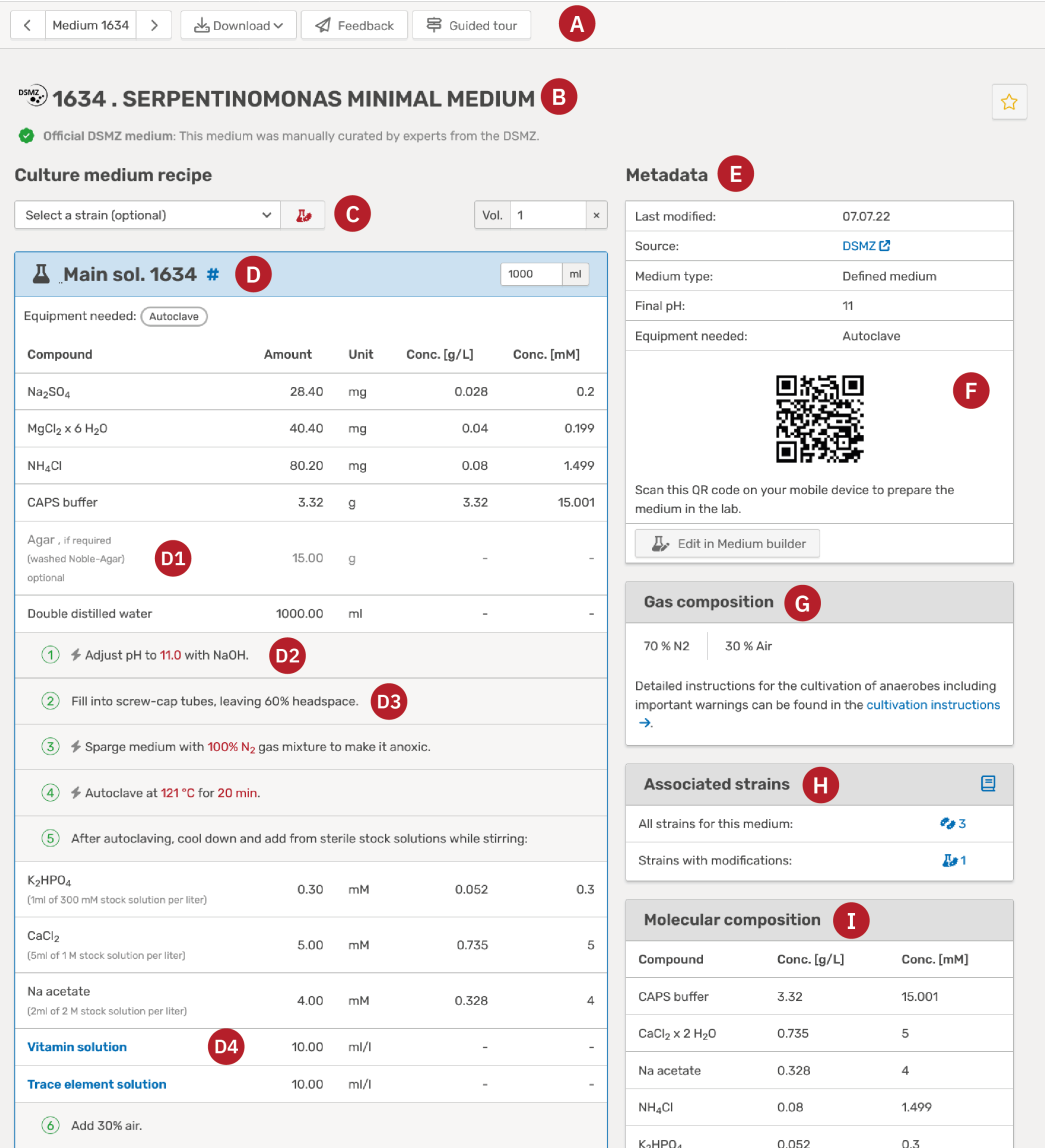
Example showing the medium viewer page for DSMZ medium 1634. (**A**) The medium toolbar, (**B**) unique number and name of the medium, (**C**) the recipe can be modified by selecting a strain or changing the volume, (**D**) the main solution recipe; all other solutions can be found on the page below, (**E**) a summary of all the important metadata, (**F**) a QR-code to move seamlessly to mobile devices, (**G**) the final gas composition for cultivation, (**H**) an overview on all associated strains with links to detail pages, (**I**) the final concentrations of all ingredients in the medium.

The core of Media*Dive* is the medium viewer. On this page, all data on a medium are integrated and displayed (Figure 1). At the very top is a toolbar (A), where the user can navigate to neighbor media, download the recipe as PDF, JSON or plain text, give feedback to the medium, or get an introduction to the page through a guided tour. Further below, the number and name of the medium the user is currently viewing are listed (B).

On the left-hand side, all solutions that are needed to prepare the medium are shown, starting with the main solution (D). Each medium has a main solution that is marked with a little flask icon. All other solutions in the recipe can be reached from the main solution, the latter thus is the best starting point when preparing a medium. Directly above are two input fields located (C). One is a strain selector, where the user can select a strain that is known to grow on the medium. As some strains might require modifications of the medium, selecting a microbial strain will lead to a strain-specific page of this medium. The other input is a volume modifier that can be used to change the volume of the entire recipe. The value must be a coefficient of 1 l, for example ‘0.5’ if only half of a liter is to be produced. The main solution also has a volume modifier that can be used to change the absolute volume in ml. However, this adjustment only affects the main solution.

Each solution is shown as a box, containing the solution name, final volume, needed equipment, other media containing the solution and the recipe (D). The latter is a table, containing all instructions, ingredients and other solutions, as well as amounts, units, and concentrations (in g/l and mmol/l, if available). Further information on an ingredient is called attribute and shown in parenthesis (D1). Ingredients can also be marked as optional (D1). Solutions that are part of the recipe are highlighted in blue and linked with their respective recipes (D4). Instructions are shown as either free text (D3) or standardized steps (e.g. for autoclaving or pH adjustment; D2).

As already mentioned, some microbial strains might need adjustments to the medium, which we call strain-specific modifications. In total, more than 3,500 strains are affected, which is why we developed a new markup to take the modifications into account. They are grouped into *addition* or *omission* of compounds or solutions, changes in *pH* or *gas phase*, or *light* conditions that are needed for growth. When selecting a strain (see above), the medium recipe is modified to take the adjustments into account: omitted ingredients are crossed out, added compounds and solutions are also added to the recipe, and the metadata, i.e. final pH and gas phase, are also adjusted.

On the right-hand side all *metadata* for the cultivation medium are located (E), starting with general information on the source, taxonomic range, final pH, needed equipment, references and descriptions. If the medium is anoxic or microoxic, the final gas phase is listed here as well (G). Next comes a summary of growth data that is available for this medium (H). First, a number of all strains from the DSMZ and JCM collections that are known to grow on this medium are displayed, with a link to a detail page that lists all strains including their growth information, modifications and external links. If modifications are pertinent, a number of strains with modifications is displayed. Another section shows related media, e.g. modified media or other media that use the same base medium as the current medium. In the metadata section, the user can also find the composition of the final medium (I). Concentrations of all compounds are given in both mass and molar composition (g/L and mmol/L, respectively; if available). The final concentrations are calculated automatically and can be downloaded in CSV or JSON format. At the bottom, an integrated list of metadata from the Bac*Dive* database for all strains that grow on this medium is provided. A link leads to an overview on the corresponding strains in Bac*Dive*.

Download of the medium recipe from the toolbar (A) is enabled in three different formats: (i) download as PDF document, which is the best choice for printing, (ii) download as JSON, which is more relevant to computationally advanced users, (iii) download as plain text in markdown format, which is most versatile as it allows users to easily transform the recipe into any format of their choice, e.g. MS Word. From these three options, currently only the PDF download will reflect strain-specific adjustments and volume changes.

Instead of printing the recipe, one can use Media*Dive* directly to prepare a medium. A QR-code can be generated in the metadata section to seamlessly move from desktop to mobile devices (F). In the mobile view, a round button with a cauldron icon is shown in the bottom-right corner. This leads directly to the *medium cooking guide*, a web page especially designed for mobile devices. On this page, all information that is not essential to prepare the medium is omitted, e.g. final concentrations. Instead, a checkbox at the beginning of each row lets users check off which ingredients they already put into the medium to keep track of the progress.

### Tools

Media*Dive* has a number of tools that support users in finding and preparing a particular cultivation medium. Aside from the search and filter options in the content pages, a medium can be searched using the medium finder. This is a powerful tool that assists the user in searching for media by defined criteria. For example, one can search for media that contain a certain substance at a specified concentration, exclude certain substances if a medium should not contain them, or search for a specific gas phase. Integration over eight search criteria is enabled.

**Figure 2.**
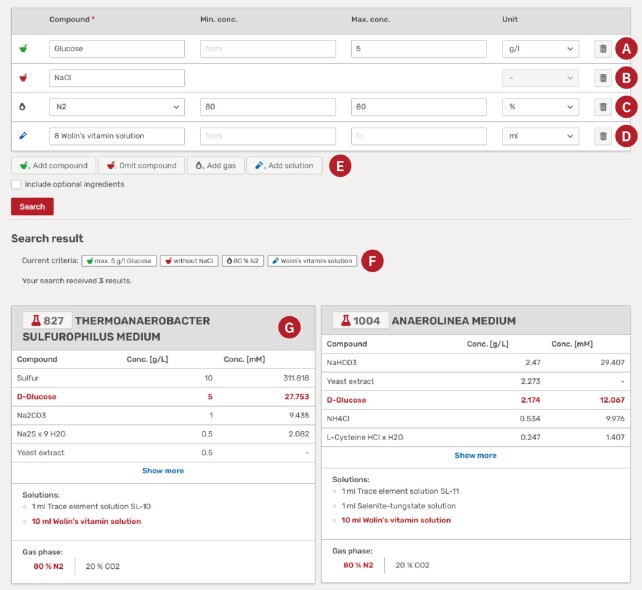
Showcase screenshot of the medium finder. Different search criteria are applied for (**A**) the presence of compounds with defined concentrations, (**B**) for the absence of compounds, (**C**) a defined gas phase, (**D**) the presence of certain solutions. (**E**) More search criteria can be added. (**F**) The search parameters are summarized. (**G**) All found media are shown in detail and matching search conditions are highlighted.

An example is shown in Figure 2, where a search for media is displayed that contain a maximum of 5 g/l glucose (A), no NaCl (B), exactly 80% N_2_ in the gas phase (C), and any amount of Wolin's vitamin solution (D). More criteria can be added via one of the buttons below (E). After performing a search, all applied criteria are displayed on top of the results (F), as well as the number of found media. Below is a list of all media where the matching criteria are highlighted (G). The solution finder works in a similar way. The user can search for compounds *per se*, but can also include absolute amounts or concentrations.

For users that want to cultivate a microorganism that is not included in the active database, the taxonomy search would be the right place to start. One can either search for a taxonomic rank (domain, phylum, class, order, family, genus, species) directly or navigate through a taxonomic tree, in which the number of media for each rank is shown. By clicking on the number, a list of all relevant media is shown on the right with the possibility to limit the results to defined media. Currently, the taxonomy search is only available for prokaryotes as it is based on the taxonomic nomenclature retrieved from LPSN (11).

Aside from previously mentioned search tools, Media*Dive* offers tools to assist in preparing media, such as a unit converter for solid and liquid compounds. Any compound present in the database with a defined mass can be converted from mass concentration to molar concentration. The same is true for liquid compounds, where the density is considered. However, one can also enter the mass or density themselves to use this tool. Other tools are the compare solution and compare media tools that assists users in deciding between two nearly identical solutions or cultivation media, respectively.

### Programmatic access

The web UI is not the only way to access the cultivation media data in Media*Dive*. Data can be retrieved using a RESTful web service (https://mediadive.dsmz.de/rest). Currently, ten end points are supported, providing general statistics, information on solutions, ingredients, and strains, as well as complete media recipes and compositions. For the comfort of the users, a detailed documentation is available. The web service is free of charge.

## USER ENGAGEMENT

**Figure 3.**
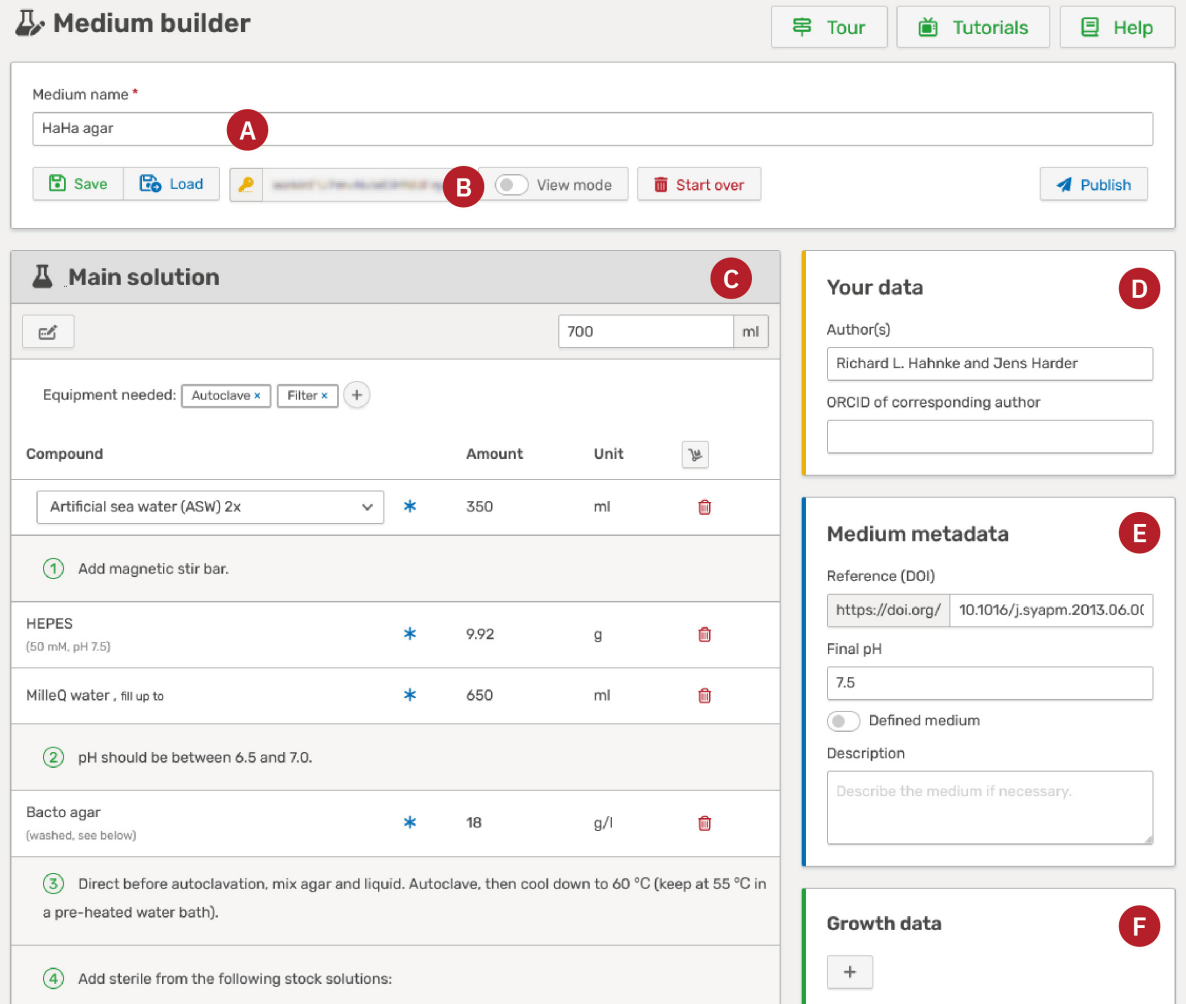
Screenshot of the medium builder. The name of the cultivation medium (**A**) can be defined in the toolbar at the top of the page, as well as various actions, such as saving the medium to the database, which will generate a unique key phrase (**B**). The key phrase can in turn be used to load and edit the medium. The recipe editor (**C**) is used to modify the recipe by adding compounds, solutions, steps, and equipment. Metadata include the authors of the cultivation medium (**D**), the medium metadata (**E**), strains that are known to grow on the medium (**F**) and the final gas composition (not shown). The shown cultivation medium was taken as a use case (20) and published under the medium number P1.

Media*Dive* is extended, curated and maintained via a comprehensive internal curation interface to provide a reliable, high-quality resource to the scientific community. To enable user engagement beyond the curated database, we integrated a lightweight editor called the medium builder. In Figure 3, the medium builder is showcased with the cultivation medium HaHa agar (20) that was designed using the medium builder and was published under the medium number P1. Users can use this interface to create their own cultivation media that will be included into the list of media and displayed on a dedicated medium viewer page. Authors of the medium will be acknowledged by the use of an Attribution 4.0 International (CC BY 4.0) license and the possibility to add both, author names (D) and a reference (E) to the metadata.

In the medium builder, users can either create a completely new medium or import an existing medium to modify it. Users can edit the recipe and metadata. For the recipe, it is possible to add ingredients, solutions (new and existing ones), instructions, and needed equipment (C). For the metadata, only the medium name is mandatory (A) but one can add a description and the final pH of the medium (E), define the gas phase, and add growth data (F). It is possible to save the cultivation medium at any time without publishing it. The user receives a unique key phrase (B) that is used to edit the medium later on. Once the medium is finished and the user submits it for publishing, it will undergo a short review process to prevent adding malicious data to the database. As soon as the medium is published, it receives a unique medium number that can be used to reference it. Furthermore, it is added to the list of media and will be visible for all users of the database. Note that this tool is currently in a test phase (beta). We included a detailed documentation as well as many tutorials and highly value feedback from our users to improve it.

## CONCLUSION AND OUTLOOK

Media*Dive* is a live-curated database of cultivation media for microorganisms of all domains of life. Our mission is to standardize cultivation media recipes and to make them freely available to the community. A modern web interface with many search, filter, and display features improves the work with cultivation media and creates new possibilities for data integration and accessibility.

Media*Dive* is committed to setting further standards for documentation and development of cultivation media in the future. This includes both the continuous expansion of the database to include new cultivation media and organisms, which is naturally interlinked with the steadily growing DSMZ culture collection. In addition but equally important, it aims at the development of new tools for the design of media to target parts of the vast majority of uncultured microorganisms. We encourage other culture collections and individual scientists to publish their cultivation media in our database to foster these efforts.

We anticipate user engagement and interaction. For the future, we will add more ways for our users to contribute, for example by adding positive and negative growth data to Media*Dive*. If our medium builder tool proves popular, we will integrate a full-fledged curation interface into Media*Dive* and import all existing user media into the new system.

## DATA AVAILABILITY

The provided data is available under the Attribution 4.0 International (CC BY 4.0) license. All data can be freely downloaded in various formats (e.g. CSV, JSON, PDF) without restrictions, except that the origin of the data has to be properly cited when used in other works.
